# The Genetic Basis of Natural Variation in Oenological Traits in *Saccharomyces cerevisiae*

**DOI:** 10.1371/journal.pone.0049640

**Published:** 2012-11-21

**Authors:** Francisco Salinas, Francisco A. Cubillos, Daniela Soto, Verónica Garcia, Anders Bergström, Jonas Warringer, M. Angélica Ganga, Edward J. Louis, Gianni Liti, Claudio Martinez

**Affiliations:** 1 Departamento de Ciencia y Tecnología de los Alimentos, Universidad de Santiago de Chile (USACH), Santiago, Chile; 2 Institute of Research on Cancer and Ageing of Nice (IRCAN) CNRS UMR 7284 - INSERM U1081, University of Nice Sophia-Antipolis, Nice, France; 3 Institute of Genetics, Queen’s Medical Centre, University of Nottingham, Nottingham, United Kingdom; 4 Department of Chemistry and Molecular Biology, University of Gothenburg, Gothenburg, Sweden; 5 Centro de Estudios en Ciencia y Tecnología de Alimentos (CECTA), Universidad de Santiago de Chile (USACH), Santiago, Chile; Simon Fraser University, Canada

## Abstract

*Saccharomyces cerevisiae* is the main microorganism responsible for wine alcoholic fermentation. The oenological phenotypes resulting from fermentation, such as the production of acetic acid, glycerol, and residual sugar concentration are regulated by multiple genes and vary quantitatively between different strain backgrounds. With the aim of identifying the quantitative trait loci (QTLs) that regulate oenological phenotypes, we performed linkage analysis using three crosses between highly diverged *S. cerevisiae* strains. Segregants from each cross were used as starter cultures for 20-day fermentations, in synthetic wine must, to simulate actual winemaking conditions. Linkage analysis on phenotypes of primary industrial importance resulted in the mapping of 18 QTLs. We tested 18 candidate genes, by reciprocal hemizygosity, for their contribution to the observed phenotypic variation, and validated five genes and the chromosome II right subtelomeric region. We observed that genes involved in mitochondrial metabolism, sugar transport, nitrogen metabolism, and the uncharacterized ORF *YJR030W* explained most of the phenotypic variation in oenological traits. Furthermore, we experimentally validated an exceptionally strong epistatic interaction resulting in high level of succinic acid between the Sake *FLX1* allele and the Wine/European *MDH2* allele. Overall, our work demonstrates the complex genetic basis underlying wine traits, including natural allelic variation, antagonistic linked QTLs and complex epistatic interactions between alleles from strains with different evolutionary histories.

## Introduction

The transformation of grape must into wine is a complex microbiological process, where the budding yeast *Saccharomyces cerevisiae* is the main species responsible for the alcoholic fermentation [1], [2]. *S. cerevisiae* wine strains are mostly diploid and homothallic, with chromosomal size polymorphisms and repetitive subtelomeric elements [3], [4], [5]. These yeasts have been selected for specific traits such as alcohol tolerance (10–14%), low concentration of residual sugar (2–5 g/L), low production of volatile acids, low nitrogen consumption, high grow rate and reproducibility of the fermentation [1], [2], [6], [7].

The genetic variability of wine yeasts have been studied at the molecular level, identifying changes in gene expression levels, ploidy and copy number variation (CNV) that serve as the main genetic adaptive signatures of the wine yeasts to fermentative processes [8], [9]. Transcriptional profiling of wine yeasts showed a generally elevated expression of genes related with stress response, sugar metabolism, nitrogen catabolism and purine biosynthesis [10], [11], [12], [13]. Similarly, array-based comparative genomic hybridization (aCGH) demonstrated substantial CNV changes, with wine yeast showing an increased copy number in genes related to the fermentative processes, such as membrane transporters, alcohol metabolism and metal resistance [14], [15], [16].

Previous population genomic surveys revealed that wine isolates form a tight phylogenetic cluster, perhaps as a result of human selection [17], [18]. The *Saccharomyces* Genome Resequencing Project (SGRP) sequenced the genome of 36 strains of *S. cerevisiae* from different geographic origins, including strains isolated from vineyards. The genome analysis resolved the population structure and described five clean lineages: Wine European (WE), West African (WA), Malaysian (MA), Sake (SA) and North America (NA) [17]. Other wine strains have also been sequenced. The genome analysis of the French commercial wine strain EC1118 identified 34 ORFs, not found in the reference genome, potentially relevant to the fermentative process such as membrane transport and carbon and nitrogen metabolism. Furthermore, part of these genes has been acquired by horizontal gene transfer or introgression from *Zygosaccharomyces bailii*, a yeast commonly found as a contaminant at the beginning of the fermentation [19]. Recently Borneman *et al.* [20] sequenced four wine strains and two beer strains, finding at least 20 new ORFs not present in the reference strain.

The expression and aCGH microarray data, as well as the genome sequence analysis, indicate that wine yeasts have unique genetic characteristics perhaps resulting from the adaptation to the fermentative process. Alternatively, most of these genetic variants are non-adaptive and were fixed by genetic drift due to population bottleneck giving rise to the Wine/European population [21]. However, one of the main challenges in biology is to understand the genetic variants that underlie phenotypic variation in complex traits. Oenological traits such as ethanol production, residual sugar after the fermentation, nitrogen uptake and volatile acidity are complex traits and are determined by multiple quantitative trait loci (QTL) [22]. The genetics mechanisms underlying these phenotypic variations can be identified by linkage analysis. This approach use crosses between two phenotypically different strains and searches for statistical linkage between the phenotype and genetic markers of the segregant strains [23]. This strategy has been fruitful in mapping many QTLs for an extensive number of phenotypes in yeast, such as: high temperature growth [24], sporulation efficiency [25], [26], cell morphology [27], DNA repair [28]. telomere length [29] ion tolerance [30] and sugar utilisation [30].

Only a limited number of studies have addressed oenological traits using linkage analysis. Marullo *et al.* [22] showed that ethanol tolerance, volatile acid production and hydrogen sulphide production are heritable polygenic traits. This allowed the mapping of a QTL for acetic acid production in chromosome IV (gene *ASP1*) [31]. Recently, Ambroset et al. [32], using a combined genetic and gene expression analysis, identified QTLs and expression QTLs (eQTLs) for oenological traits such as fermentation rates, nitrogen utilization and metabolite production. QTL mapping has been used also in Sake fermentation where 25 QTLs have been mapped for the phenotypes of higher alcohol production and aromatic components [33]. Furthermore, linkage analysis using a grid of crosses between isolates representative of the major lineages [17], allowed the detection of more than 80 QTLs for different ecologically relevant traits, indicating that the 69% of the QTLs were specific to single cross combination while 31% were strain dependent [34]. This resource of recombinant strains [34] probe a large fraction of *S. cerevisiae* standing variation, offering a great opportunity to screen for genetic variants relevant to oenological traits.

With the aim of identifying the genetic variants affecting oenological traits, we performed QTL mapping using three sets of segregants derived from crosses between phenotypically and genetically diverged strains [34]. The parental and segregant strains were subjected to 20 day fermentations in synthetic wine must and seven oenological traits were measured at the end of the fermentation. Linkage analysis allowed the detection of eighteen QTLs for different oenological phenotypes, all specific to a cross combination (context dependent) and some with pleiotropic effects. We validated six QTLs by reciprocal hemizygosity analysis in two of these crosses. The results revealed that genes related to sugar transport, mitochondrial respiration and nitrogen metabolism are key players underpinning the phenotypic differences observed in oenological traits.

## Results

### Yeast Oenological Traits Vary Quantitatively

**Figure 1:**
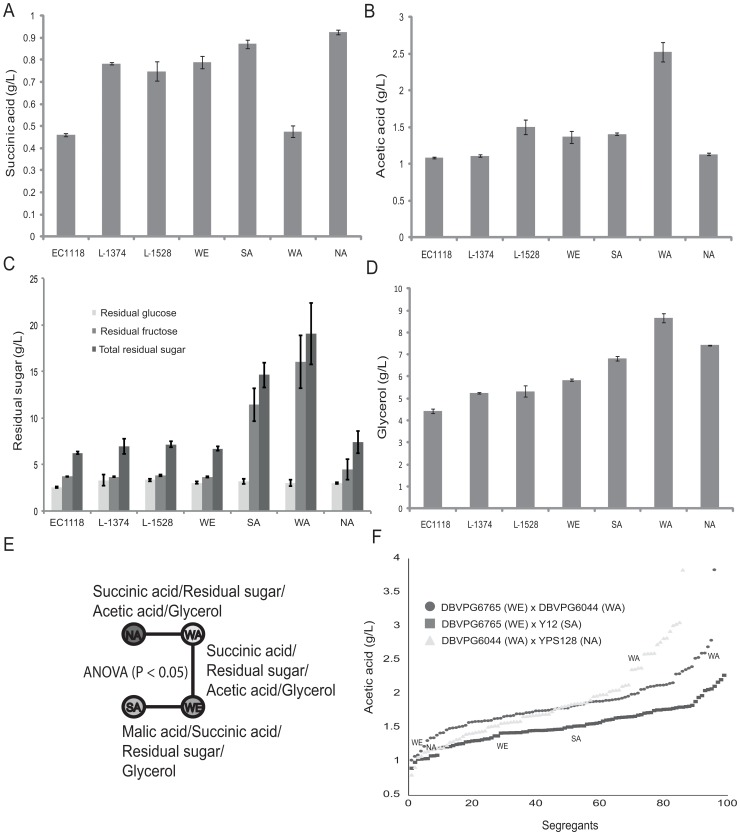
Quantitative variation in oenological phenotypes. Seven strains were phenotyped for production of key metabolites for wine making after 20 days fermentation. Three strains (EC1118, L-1374, L-1578) are used in real wine making setting. The other strains are representative of diverged genomic clusters and used for QTL mapping. (A) Succinic acid production. (B) Acetic acid production. (C) Residual sugar. (D) Glycerol production. (E) Statistical differences in the oenological phenotypes between the parental strains. (F) Example of continuous distribution in acetic acid production of segregants from three crosses.

We explored the variation in oenological properties between six parental and seven hybrids strains by measuring seven metabolic phenotypes, after 20 days of fermentation in synthetic wine must (SWM). This time point corresponds to the end of the fermentation (<6 g/L of residual sugar) using the commercial strain EC1118 as a reference (Figure 1A–D, Figure S1). The parental strains analysed were previously sequenced [17] and consist of three strains (DBVPG6765, L-1375, L-1528) belonging to the Wine/European cluster and three strains representative of genetically diverged lineages (Y12, Sake; YPS128, North American and DBVPG6044, West African) (Figure 1A–D). We found a widespread quantitative variation in all the phenotypes analysed. Interestingly, the phenotypes of DBVPG6044 strain are the most different compared to any other strain, showing high values of acetic acid production, residual sugar and glycerol production (Figure 1B, 1C and 1D). This is consistent with the general phenotypic divergence of the West African population, which remarkably low performance for a wide variety of mitotic proliferation traits [21], [30]. The phenotypic profiling of the hybrid strains shows examples of heterosis. The parent strains DBVPG6044 and Y12 showed high level of residual sugar due to their low ability in utilising fructose whereas the hybrid strain consumed a substantially larger portion of the total sugar (Figure S1C). Crosses with the West African DBVPG6044 are known to show frequent heterosis due to the hybrid masking the loss-of-function mutations that abound in its genome [21]. Based on the phenotypic properties of the parental strains we selected three crosses, DBVPG6044 (WA) x YPS128 (NA), DBVPG6765 (WE) x DBVPG6044 (WA) and DBVPG6765 (WE) x Y12 (SA), where the parental strains showed the greatest numbers of significant differences in the analysed phenotypes (ANOVA P<0.05, Figure 1E).

We used 96 segregants from each cross (total 288, from study [34]) for the SWM fermentation and analysis of oenological traits. For each segregant, SWM fermentation was carried out in two completely independent biological replicas, similarly to previous wine QTL studies [32]. We did not observe significant statistical differences between fermentation replicas (ANOVA P<0.05) and the replicas of the segregants fermentation showed very strong and significant correlations (Pearson correlation P<0.05, r >0.5 in all cases) and similar statistical values (Table S1). Phenotypic values of the segregants showed a continuous distribution, a hallmark of polygenic traits (Figure 1F, Figure S2). The phenotypic data were used to calculate the level of transgression from the parental phenotype and the heritability of the traits. The three crosses showed high values of transgression from the parental phenotype and high values of heritability, with an average heritability of 0.9 for the analysed phenotypes (Table 1). These results indicate that these phenotypes are highly suitable for linkage analysis and show the potential biotechnological application of breeding different yeast variants to generate new commercial strains.

**Table 1:** Analysis of phenotypes.

Phenotype	WA x NA	WE x WA	WE x SA
Malic acid	12% (0.9)	51% (0.9)	57% (0.9)
Succinic acid	38% (0.9)	61% (0.8)	89% (0.7)
Residual sugar	53% (0.9)	60% (0.9)	68% (0.9)
Ethanol	22% (0.8)	47% (0.8)	72% (0.8)
Acetic acid	14% (0.9)	5.3% (0.9)	75% (0.8)
Glycerol	63% (0.9)	15% (0.9)	54% (0.9)
Ammonium uptake	32% (0.8)	32% (0.8)	4.1% (0.5)

() heritability.

Percentage of transgression from parental phenotypes and heritability of the oenological traits.

### Mapping Winemaking Relevant QTLs

We performed linkage analysis for seven phenotypes in the three segregant populations. We mapped a total of 18 QTLs, four of them in the WA x NA cross and 14 in the WE x SA cross. No significant QTLs were detected in the WE x WA population. A similar set of QTLs was mapped when the two fermentation replicas were used individually for the linkage analysis, showing the high reproducibility of the fermentation process (Table 2). All the QTLs detected were specific to a single cross combination (context dependent) (Table 2). The context dependent QTLs for residual sugar (WA x NA cross) and glycerol (WE x SA) explained up to 25% of the phenotypic variance for those traits (Table 2). For two traits, residual sugar in WE X SA cross and acetic acid in WA x NA cross, the QTLs detected explain the majority of the phenotypic variation (70.8% and 60.6% respectively, Table 2). Furthermore, in the WA x NA cross one of the QTLs for acetic acid production is located in the subtelomeric region of chromosome II (Table 2). In the WE x SA cross the QTLs in chromosomes VI, IX and XV affect multiple phenotypes thus showing a pleiotropic effects (Table 2).

**Table 2:** QTLs mapped by linkage analysis.

Cross	QTL region (Chr)	LOD	Variance explained	Phenotype
WA x NA	780- Tel kb (II)*	3.95	19.5%	Acetic acid
WA x NA	201–215 kb (XIV)	3.15	15.9%	Acetic acid
WA x NA	388–479 kb (XVI)*	5.3	25.2%	Acetic acid
WA x NA	255–279 kb (XI)	2.77	14.1%	Residual sugar
WE x SA	36–77 kb (VI)*	4.4	19.0%	Malic acid
WE x SA	231–275 kb (IX)	2.8	12.6%	Malic acid
WE x SA	112–174 kb (VI)*	3.37	14.9%	Succinic acid
WE x SA	72–131 kb (IX)*	4.32	18.7%	Succinic acid
WE x SA	35–99 kb (XV)*	3.95	17.3%	Succinic acid
WE x SA	60–99 kb (XV)*	3.37	14.9%	Acetic acid
WE x SA	36–65 kb (VI)*	2.8	12.6%	Residual sugar
WE x SA	149–191 kb (IX)*	3.19	14.2%	Residual sugar
WE x SA	217–256 kb (IX)*	3.09	13.8%	Residual sugar
WE x SA	390–453 kb (X)*	3.37	14.9%	Residual sugar
WE x SA	453–511 kb (X)*	3.47	15.3%	Residual sugar
WE x SA	36–93 kb (VI)*	6.3	26.1%	Glycerol
WE x SA	153–196 kb (VI)*	3.2	14.2%	Glycerol
WE x SA	149–191 kb (IX)	2.7	12.1%	Ethanol

* Indicate QTLs detected in both fermentation replicas when linkage analysis were run separately in each replica.

The chromosomal location, LOD value, phenotypic variance explained and phenotype, are shown for the QTLs detected.

The average size of the QTLs region was 80 kb. To narrow the genetic regions mapped, we increased the marker density within these regions in segregants showing recombination within the interval, refining the QTL regions to an average size of 50 kb (Table 2). To further refine the QTL intervals in the WA x NA cross, we phenotyped 96 additional segregants obtained from a multigenerational cross (F6, [35]) of an advanced intercross line (Figure S3). We genotyped markers around the QTLs and performed linkage analysis for the F6 generation. This approach refined the QTL mapping to narrow regions (from 60 to14 kb in Chr XIV and from 70 to 24 kb in Chr XI, Table 2) facilitating the identification of causative genes.

### Dissection of the Oenological QTLs in the WA x NA Cross

**Figure 2:**
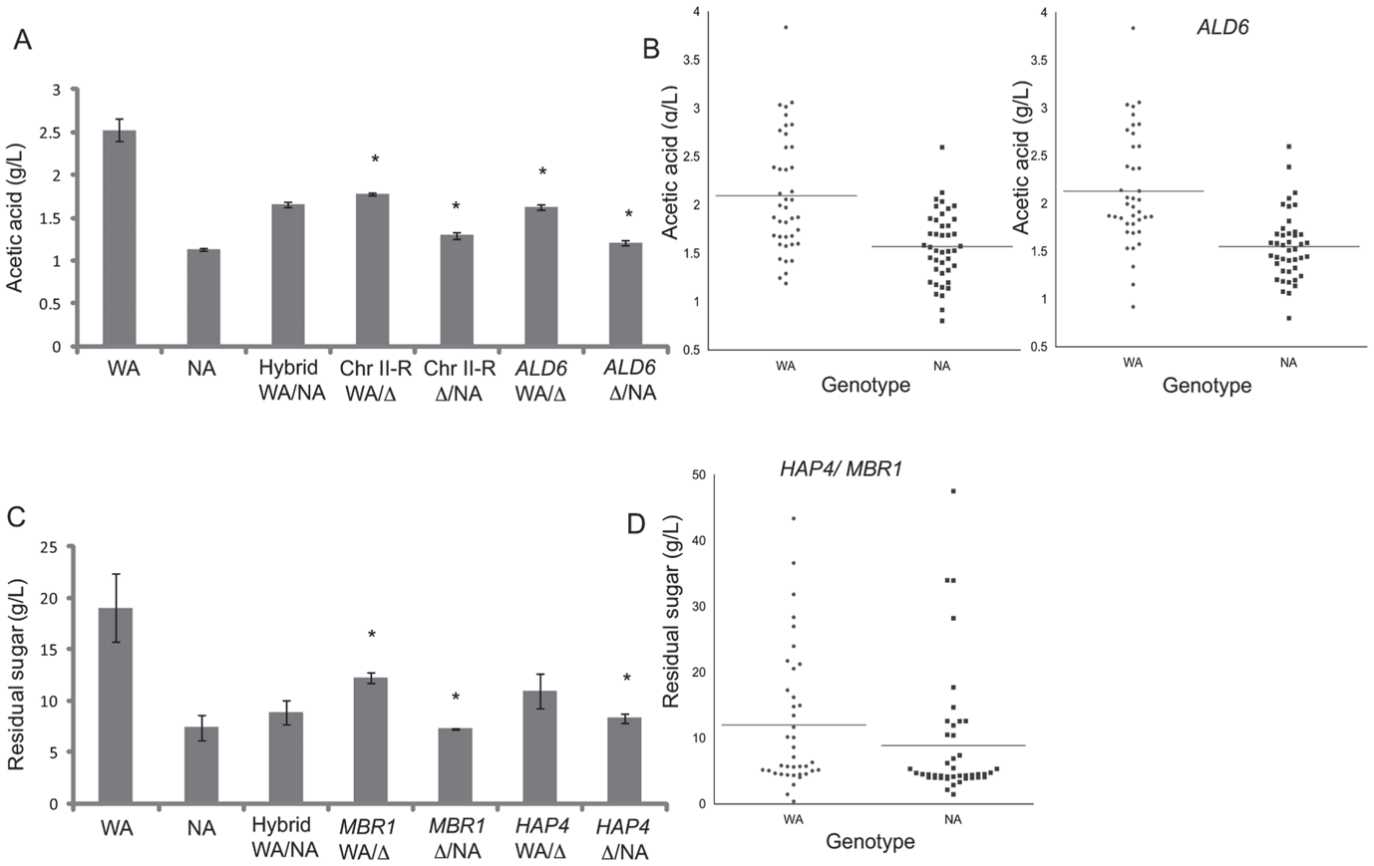
Identification of WA alleles contributing to high level of acetic acid and residual sugar. (A) Reciprocal hemizygosity analysis of subtelomeric region of chromosome II-L (Chr II-R) and *ALD6* for acetic acid production. The hybrid hemizygote strains with the WA or NA allele for Chr II-R and *ALD6* are showed. (B) Acetic acid production of segregant strains sorted for Chr II-R and *ALD6* genotypes. The horizontal lines represent the average phenotype value. (C) Reciprocal hemizygosity analysis of *MBR1* and *HAP4*. The hybrid hemizygote strains with the WA or NA allele for *MBR1* and *HAP4* are shown. (D) Residual sugar of segregant strains sorted for Chr II-R and *ALD6* genotypes. The horizontal lines represent the average of the phenotype values. (*) represents a significant statistical difference between the hemizygote strains for the same gene (ANOVA P<0.05).

We examined the genomic regions containing QTLs using sequences from the SGD and SGRP databases and selected possible candidate genes. We applied reciprocal hemizygosity analysis to the right subtelomeric region of chromosome II (Chr II-R) and 9 candidate genes (Table S2). We observed a significant allelic difference for the production of acetic acid in the subtelomeric region of chromosome II-R and for the *ALD6* gene on chromosome XVI, which encodes a cytosolic aldehyde dehydrogenase required for conversion of acetaldehyde into acetic acid. The hemizygote strains containing the Chr II-R and *ALD6* (Chr XVI QTL) alleles from WA strain showed a higher production of acetic acid (Figure 2A) in agreement with the phenotypic values of the segregant strains carrying the WA alleles for *Chr II-R* and *ALD6* (Figure 2B).

Similarly, we also validated two genes, *HAP4* and *MBR1* (Chr XI QTL), which contribute to the differences found in residual sugar. The gene *HAP4* encodes a subunit of a transcription factor required for expression of cytochromes and respiratory genes, whereas *MBR1* encodes a protein involved in mitochondrial function and its overexpression suppresses the growth defect of *HAP4* mutants [36]. The two genes are contained within the same linkage interval and are located 30 kb apart. The hemizygote strains containing the *HAP4* and *MBR1* alleles from WA strain showed high levels of residual sugar after fermentation (Figure 2C). The genotype of the segregants for the QTLs of the chromosome XI (genes *HAP4* and *MBR1*) is consistent with the phenotypic differences observed in the hemizygotes and parental strains (Figure 2D).

### Dissection of the Oenological QTLs in the WE x SA Cross

**Figure 3:**
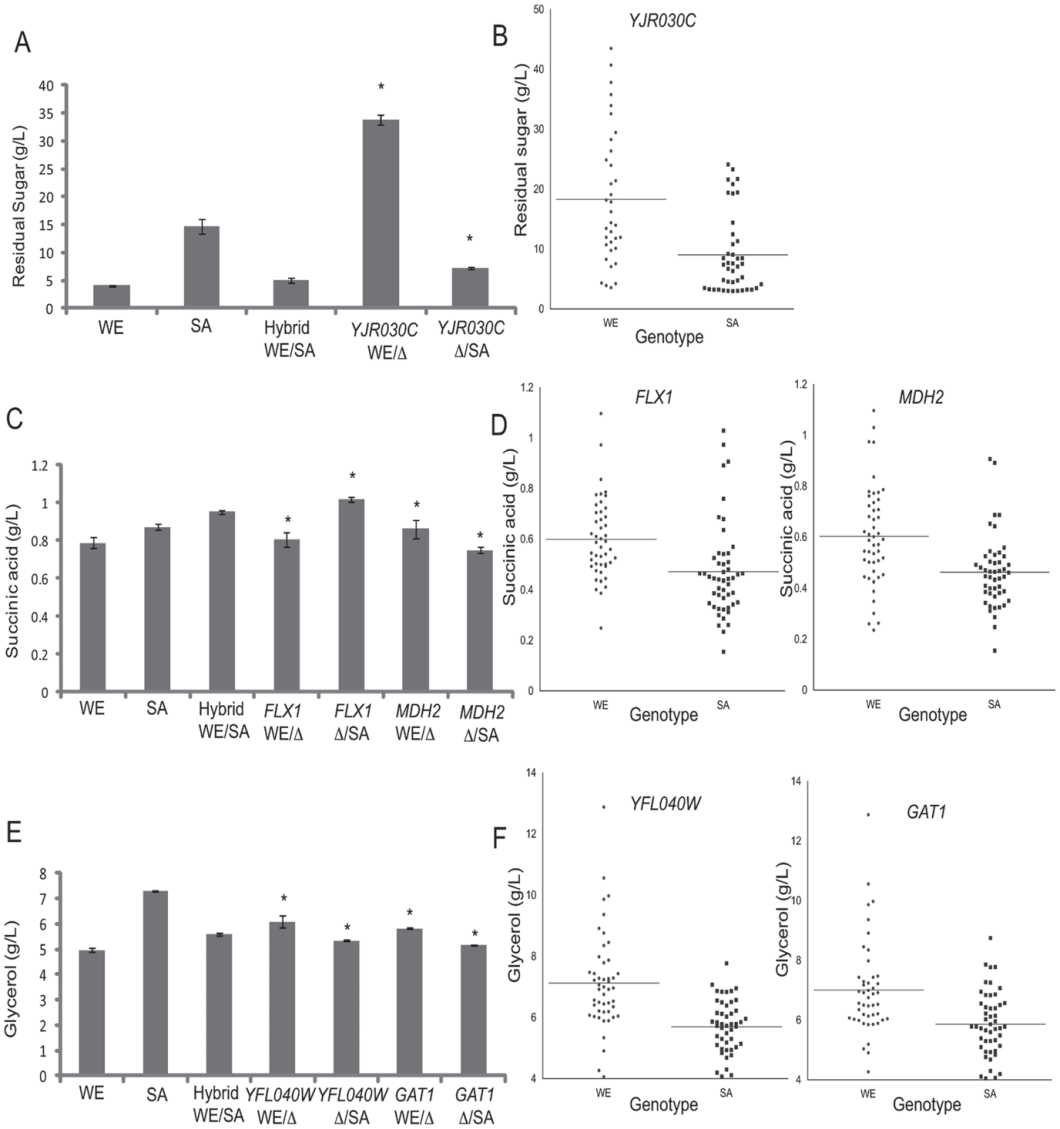
Multiple antagonistic QTLs in the WE background. (A, C and E) Reciprocal hemizygosity analysis of candidate genes for residual sugar, succinic acid production and glycerol production respectively. The hybrid hemizygote strains with the WE or SA allele are showed. (B, D and F) Residual sugar, succinic acid production and glycerol production of segregant strains, sorted by genotypes. The horizontal lines represent the average phenotype value. (*) represents a significant statistical difference between the hemizygotes strains (ANOVA P<0.05).

In the WE x SA cross, we applied reciprocal hemizygosity to 9 candidate genes (Table S2). The gene *YJR030C* encodes for an uncharacterised protein and its expression is repressed under low levels of carbon sources [37]. The hemizygote strain containing the *YJR030C* allele from WE strain has higher levels of residual sugar than the hemizygote strain with the SA allele (Figure 3A). This is consistent with overall higher residual sugar in segregants carrying the *YJR030C* WE allele and has opposite trend to the parental phenotypes indicating an antagonistic QTL (Figure 3B).

For succinic acid production we validated the effect of two genes: *FLX1* (Chr IX QTL) and *MDH2* (Chr XV QTL). *FLX1* encodes a transporter of flavin adenine dinucleotide (FAD) across the mitochondrial membrane and the activity of the succinate dehydrogenase is reduced in *FLX1* mutants [38], [39]. Gene *MDH2* codes for a cytosolic malate dehydrogenase involved in malate/oxalacetate interconvertion, glyoxylate cycle and gluconeogenesis pathway [40]. The hemizygote strain containing the *FLX1* allele from WE strain produces lower levels of succinic acid compared to the SA allele (Figure 3C). The phenotype of the hemizygote strains agree with the phenotype of the parental strains (Figure 3C); however, the genotype of the segregants show that those with the allele from WE strain produce more succinic acid than the segregants with the allele from SA strain, showing the antagonistic effect of this QTL region (chromosome IX) and suggesting the presence of additional QTLs linked to *FLX1* that masks its effect (Figure 3D). The hemizygote strain containing the *MDH2* allele from WE strain produced more succinic acid than its counterpart carrying the SA allele (Figure 3C). This is in agreement with the segregants carrying the WE allele producing more succinic acid, i.e. the opposite trend as what was observed for the parental phenotypes, indicating the presence of an antagonistic QTL (Figure 3D).

We then investigated the glycerol production in the hemizygote strains for *YFL040W* and *GAT1* (Chr VI QTL). We observed that these two hemizygotes showed significant phenotypic differences. The *YFL040W* gene is a putative sugar transporter and *GAT1* is a transcriptional activator of genes involved in nitrogen catabolite repression. We selected *GAT1* given the tight relationship between the nitrogen and sugar metabolism [41]. The hemizygote strains containing the *YFL040W* and *GAT1* alleles from WE strain produce more glycerol than hemizygote strains containing alleles from SA strain (Figure 3E). This is in agreement with the phenotypes of the segregants carrying the WE alleles producing more glycerol (Figure 3F). These linked QTLs appear to be antagonistic compared to the parental phenotype.

### Oenological QTLs Show Pleiotropic Effects and Background Dependent Haploinsufficiency

The hemizygote strains described above showed changes in other oenological phenotypes, suggesting pleiotropic effects. For example, the hemizygote strains for the *YFL040W* gene have differences in the production of acetic acid and glycerol (Table S3). In the case of the hemizygote strains for *HAP4*, *MBR1* and *YJR030C,* differences in residual sugar levels after fermentation were due to the high levels of remaining fructose (Table S3). To further investigate the role of the uncharacterized ORF *YJR030C* in the residual sugar phenotype, we measured the mitotic proliferation capacity using glucose and fructose as a carbon source of WE and SA strains carrying a deletion of this ORF. The results showed a strong defect in growth rate for *YJR030C*Δ strains compared to the wild type strains (Figure S4). Growth defect was significantly stronger in the WE genetic background, which also showed pronounced haploinsufficiency when a single gene copy was deleted in a diploid background. These results confirm a role of *YJR030C* in sugar metabolism and the role is more pronounced in the WE background.

We tested haploinsufficiency by generating homozygote diploid parental strains with a single copy of the all the QTLs validated. In the WA and NA parental strains we constructed diploid strains with one allele of the *ALD6*, *HAP4* and *MBR1* genes and the subtelomeric region of chromosome II. In the WE and SA parental strains we developed diploid strains with one allele of the *FLX1*, *MDH2*, *YJR030C*, *YFL040W* and *GAT1* genes. The fermentation and phenotypic analysis showed background dependent haploinsufficiency effects for *MBR1* in the WA background and *YFL040W* in the SA strain, affecting the residual sugar and glycerol production, respectively (Figure S5). We did not observe haploinsufficiency for *YJR030C* for the residual sugar at the end of the fermentation, consistent with results obtained from growth curves where haploinsufficiency only impact growth rate but not growth efficiency (final cell concentration, Figure S4).

### Expression Variants and Deleterious Polymorphisms Underlie Oenological QTLs

**Figure 4:**
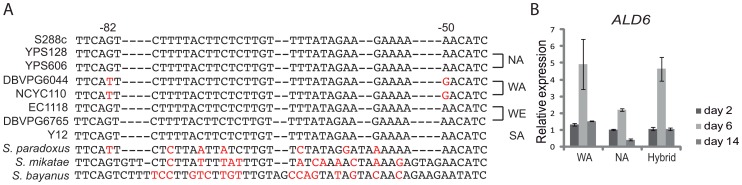
Higher expression levels of WA *ALD6* are consistent with increased acetic acid production. (A) Analysis of promoter region of the *ALD6* gene. Residue at positions −82 and −50 are evolutionary conserved in other *Saccharomyces sensu stricto* species but not in the West African lineage. Nucleotide changes with respect to the reference genome (S288c strain) are shown in red. (B) Expression of *ALD6* in the parental and hybrid strain at three different time points of the fermentation process.

We analysed the polymorphisms within the coding sequences and regulatory regions in the validated QTLs and compared them to the available population genomics datasets (Figure 4A and Figure S7 [17]). The West African *ALD6* allele (strains DBVPG6044 and NCYC110) contains two polymorphisms within the promoter region at positions -50 and -82 (Figure 4A). These polymorphisms are private to the West African and Malaysian population and occur in otherwise evolutionary well conserved position, this potentially can affect the expression level of *ALD6* gene. We measured expression levels of *ALD6* and all the other *ALD* isoenzymes (*ALD2*, *ALD3*, *ALD4* and *ALD5*) at days 2, 6 and 14 of the fermentation in the WA and NA parent strains. These results showed a 4.9 fold increase in the expression of *ALD6* after 6 days of fermentation in the WA strain, consistent with his higher acetic acid production (Figure 4B and Figure S6).

The *HAP4* gene of WA strain has four non-synonymous SNPs that are private to this lineage and occur in conserved domains (Figure S7A). These non-synonymous changes include an aminoacid change with different charge (lysine for glutamic acid; K501E) likely to impact on the protein structure. This change occurs in an extremely well conserved position, potentially making the WA HAP4p partially defective (Figure S7A).

We detected several aminoacid changes in the YJR030Cp that can account for the allelic variation in the residual sugar phenotype (Figure S7B). These changes are private to either the SA or WE lineages. In addition, the aminoacid change (F11L) is a rare variant polymorphic within the Wine/European population and occurs in an evolutionary conserved domain (Figure S7B). Sequence analysis of the ORF *YJR030C* showed no known sequence domains or motifs detectable. However, this ORF is conserved in all the *Saccharomyces sensu stricto* species, as well as in less related species as *Naumovozyma castellii*.

The *FLX1* and *MDH2* genes have single non-synonymous change in the WE strain (Figure S7C and D). For MDH2p, the aminoacid change G87C in the WE strain occur in an evolutionary conserved region and is not fixed in the WE population (e.g. EC1118). This rare variant present in the WE strain is a potential candidate to contribute to differences in succinic acid production (Figure S7D).

Likewise, we detected non-synonymous SNPs in *YFL040W* and *GAT1* genes in both the Wine/European and Sake lineages (Figure S7E and F). The sequence analysis of YFL040W protein showed a clear homology with a transporter member of the major facilitator superfamily with 12 transmembrane domains. The *YFL040W* gene is also conserved in all the *Saccharomyces sensu stricto* species, as well as *N. castellii*.

### A strong Epistatic Interaction between *FLX1* and *MDH2* Produce High Level of Succinic Acid

We experimentally tested epistatic interactions among QTLs for succinic acid and glycerol production using double hemizygote strains. The double hemizygote strains were constructed in the WE x SA cross for the *FLX1* (Chr IX QTL) and *MDH2* (Chr XV QTL) genes for succinic acid production, and for the *YFL040W* and *GAT1* genes (both in Chr VI QTL) for glycerol production (Table S3 and S4).

**Figure 5:**
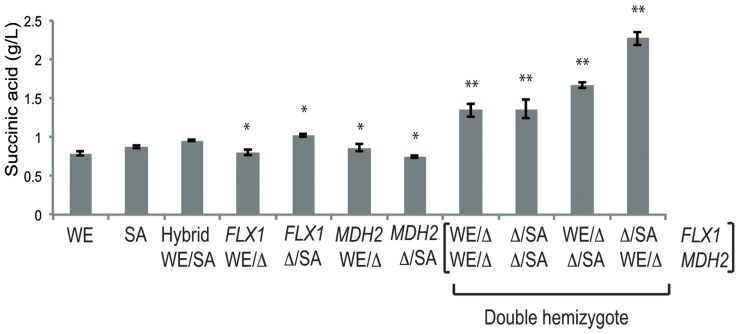
Strong epistatic interaction between diverged alleles. We measured levels of succinic acid production in parental, hybrid, and all possible single and double hemizygote combinations for *FLX1* and *MDH2* alleles in the WE and SA cross. (*) Indicates a significant statistical difference between the hemizygote strains (ANOVA P<0.05). (**) Indicates significant statistical difference between double hemizygote strains (ANOVA P<0.05).

The four possible combinations of double hemizygote strains were used for SWM fermentation. The double hemizygote strains for genes *FLX1* and *MDH2* had higher succinic acid production compared to the single hemizygote, hybrid and parental strains (Figure 5). Interestingly, the double hemizygote strain carrying allele *FLX1* from SA strain and allele *MDH2* from WE strain showed high production of succinic acid, confirming the phenotype of the single hemizygote strains (Figure 3C and Figure 5). The epistatic effect between these two alleles was measured and compared to the effect of the individual hemizygotes using an additive model [35]. The observed phenotypic value was 33 times higher than the expected value (t-test p<0.05) and an epistatic value (ε) for these two alleles of 1.6 (ε = 1.6, ε >0), indicates a positive epistatic interaction between these alleles for succinic acid. The double hemizygote strains for genes *YFL040W* and *GAT1* showed no significant differences (ANOVA p<0.05) in glycerol production and no interaction was observed between these genes (Figure S8).

## Discussion

Oenological phenotypes are complex traits and one of the challenges of yeast biology is to understand the genetic variants underlying these phenotypes. In this study, we performed linkage analysis using three crosses between highly diverged strains [34] to map oenologically relevant QTLs. We subjected over one thousand samples to 20 days of fermentation in synthetic wine must and analysed oenological traits. The parental strains showed different phenotypic profiles; especially the phenotypes of the WA strain, which are the most different compared to any other strain, confirming the population specific traits observed in this lineage [30]. We observed heterosis in the hybrid strains for different oenological phenotypes (Figure S1); this phenomenon has been previously described in yeast for heat resistance and some ecological phenotypes [24], [34] but are otherwise remarkably rare in yeast as compared to plants [21]. The segregants showed a continuous distribution a hallmark of polygenic trait [31], [33], [34]. The high levels of transgression and the high heritability of the oenological traits were similar to those previously reported [22], [42].

In total we mapped 18 QTL intervals, with some of them explaining more than 25% of the phenotypic variance. Three QTLs showed a pleiotropic effects indicating that changes in stoichiometry of proteins regulating metabolic flux impact on multiple metabolic reactions. Similar pleiotropic effect has been previously described for different stress conditions [43], [44]. The QTL intervals mapped were narrowed by increasing the genotype marker density; however, the resolution of our QTLs had an average interval size of 50–60 kb making candidate genes prioritization difficult. The use of the highly recombined F6 generation in the WA x NA cross allowed the refining of QTLs to less than 25 kb, showing the advantage of using advanced intercross lines in mapping resolution [35].

Previous studies have validated allelic variants for few wine phenotypes, some identifying the quantitative trait nucleotide (QTN) [31], [32], [33]. We applied reciprocal hemizygosity to different candidate genes and we validated six QTLs, three in the WA x NA cross and three antagonistic QTLs in the WE x SA cross. In the WA x NA cross the subtelomeric region of chromosome II and gene *ALD6* (Chr XVI QTL) explained the phenotypic variation in acetic acid production, remarking the importance of the subtelomeric regions for secondary metabolisms and confirming the role of *ALD6* in acetic acid production during the fermentation [34], [45]. In the WE x SA cross we showed that the uncharacterized ORF *YJR030C* explained part of the phenotypic variation in residual sugar, specifically the residual fructose. We further investigated the role of this gene by comparing growth curves in different carbon sources of WT and *yjr030c*Δ strains. Deletion of the *YJR030C* gene resulted in a strong growth rate defect in both WE and SA backgrounds (Figure S4), confirming indications tentatively linking this gene to carbon metabolism [37], [46].

**Figure 6:**
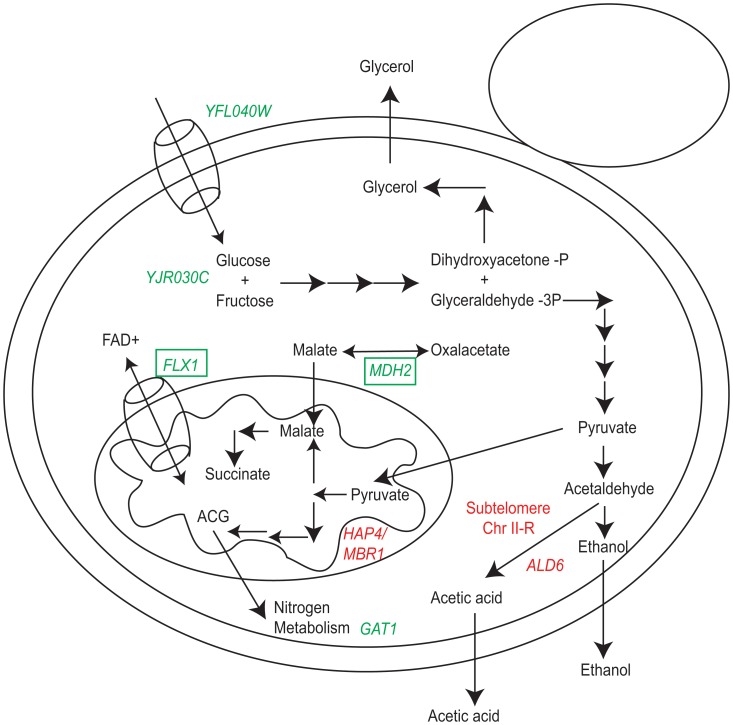
Functional role of winemaking QTLs. Genes that contributed to the natural variation in wine making QTLs are shown. Genes in red and green contribute to the natural variation in the NA x WA and WE x SA crosses, respectively. The green squares indicate genes showing positive epistatic interaction. ACG = α – ketoglutarate. Each arrow shows a metabolic step from one compound to another. See main text for further details.

To integrate the information from the linkage analysis, we propose a model on how the QTLs mapped contribute to the oenological trait variability (Figure 6). In the WA x NA cross, the *HAP4* and *MBR1* genes (Chr XI QTL) affect the residual sugar. These genes code for transcription factors that activate genes related to mitochondrial respiration. The relationship between these genes and the fermentation process is in the Krebs cycle, which is divided into the oxidative and the reductive pathway during the fermentation [41]. The oxidative pathway or fermentative mitochondrial respiration transforms pyruvate from glycolysis into α-ketoglutarate (ACG) and the reductive pathway transforms pyruvate from glycolysis into succinic acid (Figure 6). This oxidative pathway during the fermentation produces a partial mitochondrial respiration where the transcription factors *HAP4* and *MBR1* could be involved as global regulators of the expression of respiratory genes [47].

The subtelomeric region of chromosome II and *ALD6* (Chr XVI QTL) affects the production of acetic acid. The lack of subtelomeric sequence assemblies precludes a direct identification of the causative genes. For *ALD6* the relationship with the phenotype is direct, since it is involved in the transformation of acetaldehyde into acetate (Figure 6). The *ALD6* gene exhibited higher expression levels in the DBVPG6044 background and we identified two SNPs within conserved region of its promoter (Figure 4A). Linkage analysis and expression levels of all the other *ALD* isoenzymes (*ALD2*, *ALD3*, *ALD4* and *ALD5*, Figure S6) indicates that the high expression level in WA is unique to *ALD6*, confirming the key role of *ALD6* in acetic acid production [45], [48].

In the WE x SA cross, the gene *YJR030C* (Chr X QTL) affect the residual sugar after fermentation. This gene codes for a putative protein of unknown function and points toward a new role in fructose metabolism. However, no conserved domains were detected in the protein sequence of *YJR030C* perhaps consistent with a *de novo* gene birth scenario [49]. The *FLX1* gene (Chr IX QTL) affects succinic acid production. This gene is a FAD transporter across the mitochondrial membrane and mutant strains for *FLX1* show low activity of succinate dehydrogenase, an enzyme using FAD as cofactor and participating in the reductive pathway of the Krebs cycle during wine fermentation [38], [39]. The *MDH2* gene (Chr XV QTL) affects succinic acid production, interconverting malate/oxaloacetate and malate is involved in the reductive pathway of the Krebs cycle in the wine fermentation (Figure 6). The tight metabolic relationship between these genes in the reductive pathway of the Krebs cycle suggests a possible gene-gene interaction. We experimentally showed a positive epistatic interaction between the *FLX1* allele from the SA strain and the *MDH2* allele from the WE strain. This is particularly interesting because two different alleles, from parents with similar phenotypes for succinic acid production, interact in the hybrid strain to produce heterotic high levels of succinic acid (>1.5 g/L), which is detrimental to the level of acidity on the wine [50].

The *YFL040W* gene (Chr VI QTL) affects glycerol production and encodes a putative member of the sugar transporter family. Sugar transport is a key step that precedes glycolysis and during the wine fermentation allows the glycerol production (Figure 6). Similarly, *GAT1* (Chr VI QTL) is related with glycerol production since the metabolism of sugar and nitrogen are mediated in fermentation by α-ketoglutarate (ACG), a compound used for synthesis of glutamic acid, which serves as a starting point for the biosynthesis of other aminoacids [41] (Figure 6). Antagonistic linked QTLs are a common feature in the genomic landscape of yeast QTLs [51].

In conclusion our study revealed distinct mechanisms underlying the complex structure of polygenic oenological traits including allelic variation (*HAP4* and *YJR030C*), changes in transcription levels (*ALD6*), haploinsufficiency effects (*MBR1* and *YFL040W*) and epistatic gene-gene interactions resulting in heterosis (*FLX1* and *MDH2*). These findings are the first step towards understanding the genetic and molecular basis of natural variation in oenological traits.

## Materials and Methods

### Yeast Strains and Culture Conditions

The haploid or autodiploid parental strains used were isolated and generated as a part of the SGRP project [17]. The diploid commercial wine strain EC1118 was used as standard in all the fermentation experiments. The segregants utilized were derived from three crosses (WA x NA, WA x WE and WE x SA) previously described [34]. The four haploid versions (*Mat a, ho::HygMX, ura3::KanMX*) of the parental strains: YPS128 (North American, NA), DBVPG6044 (West African, WA), Y12 (Sake, SA) and DBVPG6765 (Wine/European, WE), were previously described [52]. The Hyg^r^ was replaced by Nat^r^ (*Mat a, ho::NatMX, ura3::KanMX*) as described by Janke *et al*. [53].

The F6 of the WA x NA cross was obtained by multiple rounds of random mating and sporulation, creating advanced intercross line with reduced genetic linkage se described [35]. Ninety-six segregants of the F6 were analyzed for oenological phenotypes. All the strains used and developed in this work were short term maintained on YPDA solid media (2% glucose, 0.5% peptone, 0.5% yeast extract, 2% agar) (Table S4). The sporulation of the strains was carried out in sporulation medium (potassium acetate 1%, agar 2%) for four days at 23°C.

### Fermentation and Phenotypic Analysis

Fermentations were carried out in duplicate in 50 mL of synthetic wine must (SWM) during 20 days at 28°C [16]. The SWM was supplemented with a final concentration of 0.3 mg/mL of arginine, 0.1 mg/mL of serine, 0.1 mg/mL of threonine, 0.1 mg/mL lysine, 0.1 mg/mL of aspartic acid, 0.1 mg/mL glutamic acid, 0.05 mg/mL of asparagine, 0.05 mg/mL of glutamine, 0.075 mg/mL of leucine and 0.02 mg/mL of uracil. After 20 days, the 50 mL of SWM was centrifuge at 9000×*g* for 10 min and the supernatant was filtered using 0.45 µm Millipore filter (Millipore, USA). 50 µL of filtrated must was injected in a Shimadzu Prominence HPLC equipment (Shimadzu, USA) using a Bio-Rad HPX –87H column according to Nissen *et al*. [54]. The concentration of glucose, fructose, malic acid, succinic acid, acetic acid, glycerol and ethanol was measured using the HPLC analysis. The residual sugar corresponds to the combined concentrations of glucose and fructose at the end of the fermentation. Multiple injections of the same sample in the HPLC have the exactly same readout (Table S5), therefore only one injection for each fermentation sample was required. Ammonium uptake was measured using the ammonium rapid kit (Megazyme, Ireland). The relative growth rate and efficiency phenotypes were extracted from high density microcultivation growth curves, as described in Warringer *et al*. [55].

### Linkage Analysis and Genotyping

Linkage analysis was performed as previously described [34] using the rQTL software [56] and LOD scores calculation by a non-parametric model. The significance of a QTL was determined from permutations. For each trait and cross, we permuted the phenotype values within tetrads 1000 times, recording the maximum LOD score each time. We called a QTL significant if its LOD score was greater than the 0.05 tail of the 1000 permuted LOD scores.

QTL mapping was refined by genotyping recombinant segregants as previously described [34]. Genotypes were obtained as previously described using High Resolution Melting coupled with Real Time PCR (HRM-QPCR) [29]. Genotypes information, including the additional markers genotyped in this study, is listed in table S6.

### Validations of QTLs

The genomic intervals of each QTL were examined in the *Saccharomyces* genome database (SGD) and the SGRP database. The sequence of the candidates genes were downloaded from the SGRP BLAST server of the University of Toronto (http://www.moseslab.csb.utoronto.ca/sgrp/) and the sequence alignments were obtained using the **Clustal Omega** software.

The QTLs were validated by reciprocal hemizygosity using *URA3* gene as a selectable marker with some modifications [24], [34], [57]. Briefly, we used haploid versions of the parental strains (either *Mat a*, *ho::HygMX, ura3::KanMX* or *Mat α, ho::NatMX, ura3::KanMX*) to delete each target gene and construct all possible combinations of single and double deletions (Table S4). After the deletions of the candidate genes, the strains were crossed to generate the hybrid strains and selected in double drugs plates (50 mg/mL Hygrormycin B and 100 mg/mL Nourseothricin). The diploid hybrid strains were confirmed by *Mat* locus PCR [58] and the deletions of the target genes were confirmed by PCR using the primers pairs A1/S8 or A4/S5. All the primers used in this work are listed in table S7.

### RT-QPCR Analysis

RNA extractions were carried out using the RNeasy Mini kit (Qiagen, USA). Reverse transcription (RT) reactions were carried out according to Zuzuarregui et al. [12]. The cDNA was used as a template in the real time PCR reaction (QPCR). The QPCR reactions were carried out in a final volume of 20 µL. The reaction mixture contained 10 µL of 2X Brilliant II SYBR Green QPCR Master mix (Stratagene, USA), 0.1 mg/mL of BSA (New England BioLabs, USA) and 0.25 µM of each primer (Table S7). The QPCR reaction was carried out in a LightCycler 1.5 equipment (Roche, Germany) with the following conditions: 95°C for 10 min, thirty cycles of 95°C for 30 s, 55°C for 30 s and 72°C for 30 s, a melting analysis at 95°C for 0 s, 65°C for 15 s and 95°C for 0 s with a 0.1°C/s increase in temperature, finally a cooling stage at 40°C for 30 s. Results were analysed using the LightCycler 4.0 software (Roche, Germany) and quantification of relative gene expression was done using the method described by Pfaffl MW [59] and normalized with actin (*ACT1*).

### Statistical Analysis

ANOVA statistical analyses of the phenotypes of the parental and hemizygotes strains were carried out using Statgraphics Centurion XV (Statgraphics, USA). Transgression and heritability of oenological phenotypes were calculated using the formula described by Marullo et al. [42]. The percentage of phenotypic variance explained for a QTL was calculated using the following formula:
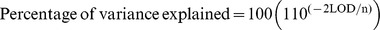
where n is the sample size.

The epistatic interaction between the *FLX1* and *MDH2* alleles was evaluated as previously described [35] and using the phenotypic values for succinic acid production of the double hemizygote strains. The positive or negative effect of the epistatic interaction between allele *FLX1* from strain Y12 and allele *MDH2* from strain WE, was evaluated using the following formula:
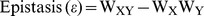
where W_X_ and W_Y_ represent the succinic acid production of two single mutant relative to the wild type, using the hybrid strain phenotype as wild type. W_XY_ represent the succinic acid production of the double hemizygote strain. Epistasis is positive when ε >0 and negative when ε <0 [60], [61].

## Supporting Information

Figure S1 **Oenological phenotypes of the parental and hybrid strains used in this work.** (A) Malic acid. (B) Succinic acid. (C) Residual sugar. (D) Ethanol. (E) Acetic acid. (F) Glycerol. (G) Ammonium uptake. (*) Significant statistical difference compared to the wine strain EC1118. (**) Significant statistical difference between the hybrid strain and the parental strains (heterosis).
(EPS)

Figure S2 **Continuous distribution of the oenological phenotypes.** The phenotypes of the segregant strains from the WA x NA, WE x WA and WE x SA crosses are shown.
(EPS)

Figure S3 **Continuous distribution of the oenological phenotypes in WA x NA F6 cross.** Ninety-six segregants were used for 20 days fermentation and analysed for the oenological traits.
(EPS)

Figure S4 ***YJR030C*** **affects the growth rate in the WE and SA genetic background.** Relative growth rate, Log_2_ (diploid WE in 2% glucose/strain), and efficiency, Log_2_ (strain/diploid WE in 2% glucose), of haploid and diploid WE and SA strains with and without *YJR030C*. Error bars = SEM (n = 4). High values = good growth, low values = poor growth.
(EPS)

Figure S5 **Haploinsufficiency is genetic background dependent.** The phenotypes of the diploids parental strains and the diploids parental strains with one allele of the candidate genes (Δ) are shown. (A and B) Acetic acid production and residual sugar respectively. (C, D and E) Succinic acid production, residual sugar and glycerol production respectively. (*) represents a significant statistical difference between the diploid parental strain with one allele of the candidate gene with respect to the wild type diploid parental strain.
(EPS)

Figure S6 **Expression analysis of the** ***ALD*** **gene family.** Expression levels of member of the ALD gene family (normalised using *ACT1*) at three different timepoint of the fermentation process.
(EPS)

Figure S7 **Protein sequence alignment of validated genes.** A, B, C, D, E and F Aminoacid changes in the proteins Hap4p, Yjr030cp, Flx1p, Mdh2p, Yfl040wp and Gat1p respectively. The aminoacid changes respect to the reference (S288c strain) are in red.
(EPS)

Figure S8 **Absence of genetic interaction between** ***YFL040W*** **and** ***GAT1*** **alleles.** Glycerol production in parental, hybrid, hemizygotes and double hemizygotes strains are showed. The hybrid hemizygote and double hemizygote strains with the WE and SA alleles for *YFL040W* and *GAT1* are showed. The asterisks (*) represent a significant statistical difference between the hemizygotes strains for the same gene (ANOVA P<0.05).
(EPS)

Table S1 Correlation between fermentation replicas in the analysed crosses. The Pearson correlation (p<0.05) and ANOVA (p<0.05) test were made between both fermentation replicas for all the phenotypes analysed.
(XLSX)

Table S2 Selection of candidate genes contributing to phenotypic variation.
(PDF)

Table S3 Oenological phenotypes of the single and double hemizygote strains. Significant statistical differences between the parental strains (green) and between the hemizygote strains (yellow) are shown (ANOVA P<0.05).
(XLSX)

Table S4 Strains of *S. cerevisiae* constructed in this work. (A) Control and parental strains. (B) Hemizygotes strains. (C) Double hemizygotes strains.
(PDF)

Table S5 Fermentation variability and reproducibility of the HPLC injections.
(XLSX)

Table S6 Genotype of the crosses used for linkage analysis including additional markers genotyped in this study. The name, chromosome (chr) and position of the markers are shown.
(XLSX)

Table S7 Primers used in this study. (A) Primers for QPCR-HRM in the DBVPG6044 x YPS128 cross. (B) Primers for QPCR-HRM in the WE x SA cross. (C) Primers for reciprocal hemizygosity in the WA x NA cross. (D) Primers for reciprocal hemizygosity in the WE x SA cross. (E) Primers for confirmation of the hemizygotes strains in the WA x NA cross. (F) Primers for confirmation of the hemizygotes strains in the WE x SA cross. (G) Primers for RT-QPCR expression analysis.
(PDF)
