# Supplementary material for: The Genetic Basis of Natural Variation in Oenological Traits in Saccharomyces cerevisiae
Source: PLoS One. 2012 Nov 21;7(11):e49640. doi: 10.1371/journal.pone.0049640 (PMC3504119; doi:10.1371/journal.pone.0049640)
Supplement: Table S2 — Selection of candidate genes contributing to phenotypic variation. (PDF) [file pone.0049640.s010.pdf]

Table S2.

Chr	Gene	Phenotype	Description
A. DBVPG6044 x YPS128 cross			
II	-	Acetic acid	Subtelomeric region.
II	*VBA2*	Acetic acid	Permease of basic amino acids in the vacuolar membrane.
XIV	*ZWF1*	Acetic acid	Glucose-6-phosphate dehydrogenase (G6PD), catalyzes the first step of the pentose phosphate pathway; involved in adapting to oxidatve stress.
XVI	*ALD6*	Acetic acid	Cytosolic aldehyde dehydrogenase, activated by Mg2+ and utilizes NADP+ as the preferred coenzyme; required for conversion of acetaldehyde to acetate; constitutively expressed; locates to the mitochondrial outer surface upon oxidative stress.
XVI	GCR1	Acetic acid	Transcriptional activator of genes involved in glycolysis; DNA-binding protein that interacts and functions with the transcriptional activator Gcr2p.
XI	GAF1	Residual sugar	Glutamine-fructose-6-phosphate amidotransferase, catalyzes the formation of glucosamine-6-P and glutamate from fructose-6-P and glutamine in the first step of chitin biosynthesis.
XI	*AAT1*	Residual sugar	Mitochondrial aspartate aminotransferase, catalyzes the conversion of oxaloacetate to aspartate in aspartate and asparagine biosynthesis.
XI	*HAP4*	Residual sugar	Subunit of the heme-activated, glucose-repressed Hap2p/3p/4p/5p CCAAT-binding complex, a transcriptional activator and global regulator of respiratory gene expression; provides the principal activation function of the complex.
XI	*MBR1*	Residual sugar	Protein involved in mitochondrial functions and stress response; overexpression suppresses growth defects of hap2, hap3, and hap4 mutants.
B. DBVPG6765 x Y12 cross			
VI	YFL040W	Malic acid/Residual sugar/Glycerol	Putative transporter, member of the sugar porter family; YFL040W is not an essential gene.
VI	GAT1	Glycerol	Transcriptional activator of genes involved in nitrogen catabolite repression; contains a GATA-1-type zinc finger DNA-binding motif; activity and localization regulated by nitrogen limitation and Ure2p
VI	HXT10	Succinic acid/Glycerol	Putative hexose transporter, expressed at low levels and expression is repressed by glucose.
IX	FLX1	Succinic acid	Protein required for transport of flavin adenine dinucleotide (FAD), a synthesis product of riboflavin, across the mitochondrial membrane

Continuation Table S2

IX	PFK26	Residual sugar/Ethanol	6-phosphofructo-2-kinase, inhibited by phosphoenolpyruvate and sn-glycerol 3-phosphate; has negligible fructose-2,6-bisphosphatase activity; transcriptional regulation involves protein kinase A
IX	RGI2	Residual sugar/Malic acid	Putative protein of unknown function; expression induced under carbon limitation and repressed under high glucose
X	PET130	Residual sugar	Protein required for respiratory growth; the authentic, non-tagged protein is detected in highly purified mitochondria in high-throughput studies
X	YJR030C	Residual sugar	Putative protein of unknown function; expression repressed in carbon limited vs carbon replete chemostat cultures; YJR030C is a non-essential gene
XV	MDH2	Succinic acid/acetic acid	Cytoplasmic malate dehydrogenase, one of three isozymes that catalyze interconversion of malate and oxaloacetate; involved in the glyoxylate cycle and gluconeogenesis during growth on two-carbon compounds; interacts with Pck1p and Fbp1
