# Supplementary material for: The Genetic Basis of Natural Variation in Oenological Traits in Saccharomyces cerevisiae
Source: PLoS One. 2012 Nov 21;7(11):e49640. doi: 10.1371/journal.pone.0049640 (PMC3504119; doi:10.1371/journal.pone.0049640)
Supplement: Table S4 — Strains of S. cerevisiae constructed in this work. (A) Control and parental strains. (B) Hemizygotes strains. (C) Double hemizygotes strains. (PDF) [file pone.0049640.s012.pdf]

Table S4.

Strain	Genotype	Source
(a)		
EC1118	*MAT a/α*	Lalvin
YPS128	*MAT a/α*	[17]
DBVPG6044	*MAT a/α*	[17]
DBVPG6765	*MAT a/α*	[17]
Y12	*MAT a/α*	[17]
L-1374	*MAT a/α*	[17]
L-1528	*MAT a/α*	[17]
YPS128 (NA)	*MAT a, ho::HygMX, ura3::KanMX*	[52]
DBVPG6044 (WA)	*MAT a, ho::HygMX, ura3::KanMX*	[52]
DBVPG6765 (WE)	*MAT a, ho::HygMX, ura3::KanMX*	[52]
Y12 (SA)	*MAT a, ho::HygMX, ura3::KanMX*	[52]
L-1374	*MAT a, ho::HygMX, ura3::KanMX*	[52]
L-1528	*MAT α, ho::HygMX, ura3::KanMX*	[52]
Cc12	Hybrid YPS128 x Y12	[34]
Cc16	Hybrid DBVPG6044 x Y12	[34]
Cc20	Hybrid DBVPG6765 x YPS128	[34]
Cc22	Hybrid DBVPG6044 x YPS128	[34]
Cc24	Hybrid DBVPG6765 x DBVPG6044	[34]
Cc28	Hybrid DBVPG6765 x Y12	[34]
L-3561	Hybrid L-1374 x L-1528	This work
YPS128	*MAT α, ho::NatMX, ura3::KanMX*	This work
DBVPG6044	*MAT α, ho::NatMX, ura3::KanMX*	This work
DBVPG6765	*MAT α, ho::NatMX, ura3::KanMX*	This work
Y12	*MAT α, ho::NatMX, ura3::KanMX*	This work
(b)		
Cc22-Tel (NA)	*subtelomero Cr II (WA)::URA3*	This work
Cc22-Tel (WA)	*subtelomero Cr II (NA)::URA3*	This work
Cc22-VBA2 (NA)	*VBA2 (WA)::URA3*	This work
Cc22-VBA2 (WA)	*VBA2 (NA)::URA3*	This work
Cc22-ZWF1 (NA)	*ZWF1 (WA)::URA3*	This work
Cc22-ZWF1 (WA)	*ZWF1 (NA)::URA3*	This work
Cc22-YNL234W (NA)	*YNL234W (WA)::URA3*	This work
Cc22-YNL234W (WA)	*YNL234W (NA)::URA3*	This work
Cc22-ALD6 (NA)	*ALD6 (WA)::URA3*	This work
Cc22-ALD6 (WA)	*ALD6 (NA)::URA3*	This work
Cc22-GCR1(NA)	*GCR1(WA)::URA3*	This work
Cc22-GCR1(WA)	*GCR1(NA)::URA3*	This work
Cc22-GAF1 (NA)	*GAF1 (WA)::URA3*	This work
Cc22-GAF1 (WA)	*GAF1 (NA)::URA3*	This work
Cc22-AAT1 (NA)	*AAT1 (WA)::URA3*	This work
Cc22-AAT1 (WA)	*AAT1 (NA)::URA3*	This work
Cc22-HAP4 (NA)	*HAP4 (WA)::URA3*	This work
Cc22-HAP4 (WA)	*HAP4 (NA)::URA3*	This work
Cc22-MBR1 (NA)	*MBR1 (WA)::URA3*	This work
Cc22-MBR1 (WA)	*MBR1 (NA)::URA3*	This work

Continuation Table S4

Cc28-YFL040W (WE)	*YFL040W (SA)URA3*	This work
Cc28-YFL040W (SA)	*YFL040W (WE)::URA3*	This work
Cc28-GAT1 (WE)	*GAT1 (SA)URA3*	This work
Cc28-GAT1 (SA)	*GAT1 (WE)::URA3*	This work
Cc28-HXT10 (WE)	*HXT10 (SA)::URA3*	This work
Cc28-HXT10 (SA)	*HXT10 (WE)::URA3*	This work
Cc28-FLX1 (WE)	*FLX1 (SA)::URA3*	This work
Cc28-FLX1 (SA)	*FLX1 (WE)::URA3*	This work
Cc28-PFK26 (WE)	*PFK26 (SA)::URA3*	This work
Cc28-PFK26 (SA)	*PFK26 (WE)::URA3*	This work
Cc28-RGI2 (WE)	*RGI2 (SA)URA3*	This work
Cc28-RGI2 (SA)	*RGI2 (WE)::URA3*	This work
Cc28-PET130 (WE)	*PET130 (SA)::URA3*	This work
Cc28-PET130 (SA)	*PET130 (WE)::URA3*	This work
Cc28-YJR030C (WE)	*YJR030C (SA)URA3*	This work
Cc28-YJR030C (SA)	*YJR030C (WE)::URA3*	This work
Cc28-MDH2 (WE)	*MDH2 (SA)::URA3*	This work
Cc28-MDH2 (SA)	*MDH2 (WE)::URA3*	This work
(c)		
Cc28- FLX1 (WE)/MDH2 (WE)	*FLX1 (SA)::URA3, MDH2 (SA)::URA3*	This work
Cc28- FLX1 (SA)/MDH2 (SA)	*FLX1 (WE)::URA3, MDH2 (WE)::URA3*	This work
Cc28- FLX1 (WE)/MDH2 (SA)	*FLX1 (SA)::URA3, MDH2 (WE)::URA3*	This work
Cc28- FLX1 (SA)/MDH2 (WE)	*FLX1 (WE)::URA3/MDH2 (SA)::URA3*	This work
Cc28-YFL040W (WE)/GAT1 (WE)	*YFL040W (SA)::URA3, GAT1 (SA)::URA3*	This work
Cc28-YFL040W (SA)/GAT1 (SA)	*YFL040W (WE)::URA3, GAT1 (WE)::URA3*	This work
Cc28-YFL040W (WE)/GAT1 (SA)	*YFL040W (SA)::URA3, GAT1 (WE)::URA3*	This work
Cc28-YFL040W (SA)/GAT1 (WE)	*YFL040W (WE)::URA3, GAT1 (SA)::URA3*	This work
