# Supplementary material for: The Genetic Basis of Natural Variation in Oenological Traits in Saccharomyces cerevisiae
Source: PLoS One. 2012 Nov 21;7(11):e49640. doi: 10.1371/journal.pone.0049640 (PMC3504119; doi:10.1371/journal.pone.0049640)
Supplement: Table S7 — Primers used in this study. (A) Primers for QPCR-HRM in the DBVPG6044 x YPS128 cross. (B) Primers for QPCR-HRM in the WE x SA cross. (C) Primers for reciprocal hemizygosity in the WA x NA cross. (D) Primers for reciprocal hemizygosity in the WE x SA cross. (E) Primers for confirmation of the hemizygotes strains in the WA x NA cross. (F) Primers for confirmation of the hemizygotes strains in the WE x SA cross. (G) Primers for RT-QPCR expression analysis. (PDF) [file pone.0049640.s015.pdf]

Table S7.

Primer	Sequence 5´ - 3´
(a)	
SPO23_FW	TCATGCCCAATGAAAGAGTG
SPO23_RS	TGGCCTAACATAGATGCCAAA
BIT2_FW	GCGCTTGCGGTACTATATCA
BIT2_RS	GGTCAAAATTAAAGGGAGAAAGA
SNF5_FW	AGGCATCATGTGCAAATACAA
SNF5_RS	CTGAACATTGGTGGGAGGTT
PCA1_FW	TTAATGAGGAATGGCCACAA
PCA1_RS	GAGAATCGGGATCGTCAATA
NAN1_FW	CATTCAAAACGTGCAAATGG
NAN1_RS	TGAAGCTTTTTACGAAGTGTCTG
FMP30_FW	ACCCAGGGTCTTGTGGATAA
FMP30_RS	ATTGCCGCTAGAATGCAAAG
RPS9A_FW	TGGCAAATTTTTCATCGAGAG
RPS9A_RS	CCGATCCTCAAAATCATAGCA
NOP4_FW	ACCAATCCCGATGACAAAAA
NOP4_RS	AGTTTCTCGCGCTTCTCGTA
CSL4_FW	CGCTACAGAAAAGCGCAAAT
CSL4_RS	TTTAATCACGTTCCCGCTTC
PSY2_FW	GCTTTTCTCAAGACATCACCA
PSY2_RS	TTTTTAAACTCTTCCAGGAGAACA
PEX27_FW	TTTAGCAACCTGAAAACTGACA
PEX27_RS	AATTTTGCTTGGCAATTTTTCTT
RFC1_FW	TGACTTTGATTTCACACCCCTA
RFC1_RS	CATATGGCCGGCAACTTTT
HES1_FW	TTCAGGAAGGGGGTATTTCA
HES1_RS	GAGGATGTTGTTCTTCGATTGG
NAT5_FW	TTAGCGAAGCTTTTTCACAAAT
NAT5_RS	TTCGAAGATGGCTTTTTATTTT
YPK1_FW	GGAGCCACTAGTTTTCCCAGA
YPK_RS	TCCCTAGTGAATTCCTCATCG
KTI12_FW	GAGAATATGTCCAAGCCAAGC
KTI12_RS	AAAAAGGACACAATTTGGGAAA
RRP14_FW	AAGTGGCCCTTGCACCAT
RRP14_RS	ATTCTTGCTCAAAGGAAACG
LHS1_FW	TCGTTTAAGACCAAGCCGTTA
LHS1_RS	TCTTTATAGCTCCTGAATTAACTGTGA
UBX7_FW	GGTGATGCATGAGCAGATGA
UBX7_RS	AAACTCGGGGCTATCTGTGA
TFB2_FW	GGATTTTAGCGAGGGGAGTG
TFB2_RS	AGCCTGAGGAACGCATTAAC
NAT52_FW	TCCATCGGTTCAAAATTACTG
NAT52_RS	AAATTTTTCAGCCATCTGTTAAGAA
VMA5_FW	TGTCGACACAGAGTATGAACCA
VMA5_RS	GGAAGGATTGGAACTCAACG
MGE1_FW	TTTTTCTTTCTCTGCCTGGTTT

Continuation Table S7	
MGE1_RS	AATGGAATTGACCAGTCCTCA
(b)	
FET5_FW	CAATGCGCAGTTATTGAAACA
FET5_RS	TTCGTTATAAGGGACGGTCA
FET5_FW	GGCGAGGTTATCCCTAACGA
FET5_RS	GGGAATGGAATGAACAAAATG
AGX1_FW	ATTGCCGGAGGTATTCACAA
AGX1_RS	TGCAAGAATTAGAAAAGCCATAA
AGX1_FW2	ACCGCTTGCAACAAGAATTT
AGX1_RS2	ATCCTGGGGATGGTTTCATT
HXT10_FW	CGGATGTGGGGTACACTCAG
HXT10_RS	GTCATTGAGGCGCAGAGC
HXT10_FW2	AAAAATAACAGCGAGGATGGTT
HXT10_RS2	TGCAATAATGGGTTTGTATGGA
MSH4_FW	AGGAATGTCCTGGAAGTGTG
MSH4_RS	TGCAAGAGTCTGCAATGACA
GSY1_FW	GATCCGGGTACACTCAAAGG
GSY1_RS	AACCAAAGAAACAGAACCGAAA
GSY1_FW2	ACCCCATTCTCTTCCAATCT
GSY1_RS2	AACAAACGTTTCTGGGTTCG
TMA108_FW	TGGCTCGTCTGCTACAAACA
TMA108_RS	GGAACTGGCATTATTCGGATT
TMA108_FW2	TTGAATCAACATGGCGGTTT
TMA108_RS2	ATCTATTACAGGGCCCACGTT
TAO3_FW	CCCAAACTAATCTGGATAAACCAA
TAO3_RS	TGAACACTGCCAAGAGGCTA
MET18_FW	GGCCTTCATGAGTGAGTAGCC
MET18_RS	TCCGCTTTTTCCCAGTTAAA
POR2_FW	AAAATCTCTCAGGGCGAGAA
POR2_RS	TTTGTTACCAGATACGTACCAGATTC
POR2_FW2	TCTTGATGGATTGGGGAGTC
POR2_RS2	TTTTACTCTGAAGGCGAAGCA
SLM1_FW	GTGGAAGGAACTGGTTGTCC
SLM1_RS	TCAGCACCTGCATAACTTGC
SHQ1_FW	TCATCCTGTGTTATAGAGAATCTTGG
SHQ1_RS	TCGCAAAAATCAGCTGAAAA
ZPS1_FW	TCAGTGCATTGTATACGTGTCG
ZPS1_RS	TTTGTTCTTTTCAGGAACACAGTT
ZPS1_FW2	TCAGTGCATTGTATACGTGTCG
ZPS1_RS2	TTTGTTCTTTTCAGGAACACAGTT
TRM13_FW	CGGTTTGGGCAGATCAATTA
TRM13_RS	GGATTCTCCCTCAAACACCTT
TRM13_FW2	GAGAATCCATGGACGAGCTG
TRM13_RS2	CAGACTGACATAGCGCGAGA
RRI2_FW	ACGTTAACGATGTCGCGATT
RRI2_RS	GTTAGAGGCCAGTTTGCACAG
MSN1_FW	TTTGTAAAAGCCGTTCGAAGA
MSN1_RS	AGCAAGGGGCTTTGTATAATGA
ITR2_FW	TCAACAATTCACAGGTTGGAA
ITR2_RS	TATAACCAGCGCCACAGTCA

Continuation Table S7

IZH4_FW	TTGCTTCTGATCCTTCTGTCAA
IZH4_RS	GAGCCTTAGACTTTTCGTATTTGG
SEC28_FW	ACTGTTTCCGGTGACAACGA
SEC28_RS	AGCTCGACAATACCCTGAGC
SEC28_FW2	ATTGGGATTGCTCAATTTGC
SEC28_RS2	AATGCATGTTCGTGATCCAA
MAM33_FW	CCAATTCGCGTTGGTTTTAT
MAM33_RS	GCAAAAACCTCAAAACTTCAAAA
FIS1_FW	GCAATGTTGCTGTGACAACC
FIS1_RS	AAAAATCAGCACATACGTACATACA
RPL34B_FW	CTTGAGTGGGATTGCTCCAT
RPL34B_RS	GCATGACATTAAAACATTAGACAGC
RPL34B_FW2	TCGACCAAGGTCTTCCACTT
RPL34B_RS2	TTAAAAGGCGAACGAGTGCT
RPC17_FW	CCCTTTTAATGTAATGGATTGCTC
RPC17_RS	AGTCTTGAATATCCGGCACAA
RPC17_FW2	TTAGAATGCATGCTCTTAGTAAGTATG
RPC17_RS2	GGTTGCAGGTTCAATTCCTG
YJR008W_FW	GTACGTCGGAAGCAAGGAAG
YJR008W_RS	ATGGTGTTTCCGGTGATTTC
RAV1_FW	TTTTGGCTTCTGTGGCTTTT
RAV1_RS	CTTCAAATTCCCTCGCTCAA
RAV1_FW2	AAAATAGCACAACGGCTTGG
RAV1_RS2	GGATGTGTCTTGGGCATTG
URB2_FW	TCTGCAAAAATATACTCCAACTTGT
URB2_RS	CAAGATTATAACGACCGGACA
URB2_FW2	AAGGGGAATTGTTTCAGATTCA
URB2_RS2	GGCAAAGGAGCCTGTGAGT
YJR054W_FW	CATAATAGGTGTAGTTGGGTGGAA
YJR054W_RS	CCCGCTATAGCCTCCCATA
YJR054W_FW2	TCGCCCTTTTTGTTTCTGAC
YJR054W_RS2	TCAAAGAGATATTTCCCGTTTTGT
(c)	
TELCHRII_FW	GAAACTGAAATTTCAAACATAAACACCAAAACAAAGCATCATC AAGGGAACATATAGTAAAGAACTACACAAAAGCAACAAAGCTT TTCAATTCAATTCATCAT
G205	CTCACTATAGGGCGAATTC
VBA2_FW	TTTCTAATGTCTTTTTGTCCGGTTTTGACGGAACCGTTACAGC TTCGACGTACCAAACGATTGGTAACGAATTTAATCAGAAGCTT TTCAATTCAATTCATCAT
VBA2_RS	GTTTAGTGTCAAGTAGAATATCTAGTTTCAATACTGTTAATGTA TCAATTGAGTCGGTCAAGGGCATAGGTATATATTGTTAGCTTT TTCTTTCCAATT
YCR061W_FW	CTTGTCTATTGTATAATTCGCTAGTATTTGTTTTGCATTGTACT CTTAATACCCCAACCAAAAACAAAATAGTGAGAGTAAAGCTTT TCAATTCAATTCATCAT
YCR061W_RS	AAAGTAAAGCATATAAGCTTCTCACTCAAAGATGAGGGGGGG GTGAAAAAAAAAAGAAAAAAGAAAAGCCATTATGGCGGTAGCT TTTTCTTTCCAATT

Continuation Table S7

ZWF1_FW	AGAGTGAGCTTGCAAGATAAAATCACTCGAAAAAAAAAATTTC<br>AGTGACTTAGCCGATAAATGAATGTGCTTGCATTTTTCAGCTT<br>TTCAATTCAATTCATCAT
ZWF1_RS	TTCTCCCCCTTCCCCCTCTCCAATTGGCTGTATAGACAGAAAG<br>AGTAAATCCAATAGAATAGAAAACCACATAAGGCAAGAAGCTT<br>TTTCTTTCCAATT
YNL234W_FW	TATATGAGTCTGCCCCTCAAGGGACTCGCCACCGGACCGCTG<br>CAGTTGTTTGCATTCATCCACATATAAGCTGGTTGTGTAAGCT<br>TTTCAATTCAATTCATCAT
YNL234W_RS	GGGTAATAAAGAACAGGCAGTGATCTAAAGAATATATAATATA<br>ACCTGGGAAACTTATACACTTAATGGTACGGTTGTAGTAGCTT<br>TTTCTTTCCAATT
ALD1_FW	CTTTTACTTCTCTTGTTTTATAGAAGAAAAAACATCAAGAAACA<br>TCTTTAACATACACAAACACATACTATCAGAATACAAAGCTTTT<br>CAATTCAATTCATCAT
ALD1_RS	ATTTATTTATGACGTAAGACCAAGTAAGTTTATATGAAAGTATT<br>TTGTGTATATGACGGAAAGAAATGCAGGTTGGTACATAGCTTT<br>TTCTTTCCAATT
GCR1_FW	ACAAACCACAGTTAATAGCGAGAAAGTCGGCAAGTGACAAAC<br>GACGGTTATATTTAATTGGCCTTTGATATATATTGAAAAAGCTT<br>TTCAATTCAATTCATCAT
GCR1_RS	AATAACCTAATGACATTTCTCGTTATCGTCGCTTCGTTATTTTG<br>TTGAAGGAACTATTGTCGCGGACAACCTCAATAAACTAGCTTT<br>TTCTTTCCAATT
GFA1_FW	AAAAATTAAAAGCGAAGGAGAAGTGATTGTAGAAAGACGGAT<br>GGGAGGCTGGGGGACGAAGAGAAAGTAAAAGGGTTAATTAG<br>CTTTTCAATTCAATTCATCAT
GFA1_RS	GTATTTCTTTTCTTATATAGTTATCAGGGCCAAATAAGCAATAA<br>GTTCTCGAGCAAATCAACAGCAACTTTTATAAAAAAAAGCTTTT<br>TCTTTCCAATT
AAT_FW	TTGAACGATAGCATACTATTGAGTGCGTTTTCCCTTCTCATTTG<br>AAAATTACTATCCGTTTTTCACTGCCGAAAGACTTGAAGCTTTT<br>CAATTCAATTCATCAT
AAT_RS	CTATAAATTATTGTGTGGCAAAATGAGAAGGGAAAAGTAATAA<br>AAAAATAAAGACAAAGGTGAACTGTAAGGTGAAAAAATAGCTT<br>TTTCTTTCCAATT
HAP4_FW	TACATCAAAGAGCATTTTAATGGGTTGCTGATTTGTTTTACCTA<br>CATTTTCTAGTACAAAAAAAAAACAAAAAAAGAATCAAGCTTTT<br>CAATTCAATTCATCAT
HAP4_RS	TAATAAGAATTTTTTATTGTATAATATCATTTTGTTTTCGTGATT<br>TTTAGTTGTTTTCGTTTTATTGCAACATGCCTATTTAGCTTTTTC<br>TTTCCAATT
MBR1_FW	TTATCGATTAATGTGTTCTGAACTCGAGTAAAAGAACAGGTGT<br>GTGTGCTAAGTAAGAAGAATTATTACATTTACCCAACAAGCTTT<br>TCAATTCAATTCATCAT
MBR1_RS	AACGAACAGATGGCGATCCAGAATGTGGAAATAAATAACAAA<br>GTTGAACATCATACGGTTATTCCAAGGTGGCGAGAATGTAGCT<br>TTTTCTTTCCAATT

Continuation Table S7
(d)

YFL040W_FW	CAATAAGTGTAATATGAGAAGAGAAAAAAGAAAGAACAAGTAC AGTTTCTAACATTATACTTTAAAAATTATTAAGCACAAAGCTTTT CAATTCAATTCATCAT
YFL040W_RS	AAAAGGAATATATATACGAAAAACTATTATATTTATATATCATA GTGTTGATAAAAAATGTTTATCCATTGGACCGTGTATAGCTTTT TCTTTCCAATT
GAT1_FW	GCACTATGAGTCGCACACTTGCGGTGCCCGGCCCAGCCACAT ATATATAGGTGTGTGCCACTCCCGGCCCCGGTATTAGCAAGC TTTTCAATTCAATTCATCAT
GAT1_RS	TACATAGAGTTTGATTAGTATGTGTATATATATATGAAGCGGAC ATGGAAAGAAGCGAGTACTTTTTTTTTTTGGGGGATCAGCTTT TTCTTTCCAATT
HXT10_FW	ATTTCTTTCTCGTAATAAAAATACAATAAATAATTAATAATAATA TAGTTGATCGATATAACAAAAAAATAACAGCGAGGAAGCTTTT CAATTCAATTCATCAT
HXT10_RS	ATCTCCATGAAATAGAAGTAAAGCATCAATCCATTATCTGTTGT TATTTCTGTTTACCAGAGTACAAGAATATTTAGAAATAGCTTTT TCTTTCCAATT
FLX1_FW	AGTAGCTTTCATAGGAATATGTACAATATCTTGTCTTTGTACTT TGCAAAACCAGCACGCAGCAGGAAGCTCTTGATAATAAGCTTT TCAATTCAATTCATCAT
FLX1_RS	CCCCTTTTTCTTTCGTACTTTTTATATTTTTATCGTTTGATATTT ACAAGAAATTAAATCTATCATATAGCCTTTATTTACAGCTTTTT CTTTCCAATT
PFK26_FW	TTTTATCACTTCATTAGTCTATTTGAATAATTCATAAAATATTTC TGATTTTTTTTTTTTTTTTTTTTTTTCATATTATTTAGCTTTTCAA TTCAATTCATCAT
PFK26_RS	GCTAAAAGAGTAAAAATAATAACAAGAGGCGTAGAAGAAGGG TTTATTCAAGGGAACAAAGGCAAGAGGAATAAAAGCTCAAGCT TTTTCTTTCCAATT
RGI2_FW	CATATAAATTATTTTATATAGCATGCTATATTTCGTAAATTCATA AAATTTCGTTAATTCATAAAAACAGCTCCCCAAACTAGCTTTTC AATTCAATTCATCAT
RGI2_RS	GATTCACCTATCTGTCGAAACACGATTAAGTGCAAACGAAACA ACGTACAGTATATAACAAAGTATTTTAAATAATAAGAAAGCTTT TTCTTTCCAATT
PET130_FW	GCACATTCGATGATAAATATTTAAAACGTATATGTAGATACATA GTGGACAGCAAGAGAGATGCATGGTTGGCCCATTTCTAGCTT TTCAATTCAATTCATCAT
PET130_RS	GGAAGAAATACGGAAAACATAGAAAGGCGATGGCAGATGAAG AAAGAAAAGATTAAGAAGGTTAATAGAAAAACCAAATCAAGCT TTTTCTTTCCAATT
YJR030C_FW	GAGGAGAACTTCTAGTATATTCTGTATACCTAATATTATAGCCT TTATCAACAATGGAATCCCAACAATTATCTCAACATTAGCTTTT CAATTCAATTCATCAT
YJR030C_RS	AATTAAAATTCATCATGAACCAAGTAAAAGTTCCTCTAATTACG AACGAGCAAGCAAATTAGTATTGTGTGGGAGACGGGAAGCTT TTTCTTTCCAATT

Continuation Table S7

MDH2_FW	CATTCTTTTCCTTTTCCTACGACTGGCTTAACGGAATATTATCAATTTGCTGCATTCTTATGCTTCGGTCCGATGCTCATAGCTTTTCAATTCAATTCATCAT
MDH2_RS	GTACAGTCAGAAGTAGTCCAGAATATAGTGCTGCAGACTATTACAAAAGTTCAATACAATATCATAAAAGTTATAGTAACAAGCTTTTTCTTTCCAATT
(e)	
S5	CCTTTTGATGTTAGCAGAATTGTC
S8	CCTCTAGGTTCCTTTGTTACTTCT
TELCHRII_A1	CTTGAAGGAGGGAGCTGTTG
VBA2_A1	AAATGAAACATTAATTCCAAATCAGA
VBA2_A4	GGGGGACTTTACAGCAAGTTT
YCR061W_A1	GACGAAGCACGGCAAATTA
YCR061W_A4	TGTGACTGCAGTGAATAGGATTG
ZWF1_A4	TTGCACCCGTGTACATAAGC
YNL234W_A1	TTTTGAGATGGACGGCTGAT
YNL234W_A4	GGATTCGCATGGCTTTATTG
ALD1_A4	GGCACAAGCCTGTTCTCTCT
GCR1_A1	GAGGGGAAAAAGTGCAGTAAC
GCR1_A4	AAAATTTCGGATAGCCCATCT
GFA1_A1	CTATTCCTGCGGGTAAAACG
AAT1_A1	TGATGAAATTCCCTACGAGGA
HAP4_A1	CCTTACGGGATAACCTCTGC
HAP4_A4	TTTTTATGTGCCGGGAGTTC
MBR1_A4	CCAAACTCGCAAGGTAAAGG
(f)	
YFL040W_A1	TACAACCGTTCCCTGAGAGG
YFL040W_A4	GGTCCATCTATCGTTCACCA
GAT1_A1	GCGCCATAATTAAAACCTAGC
GAT1_A4	TTAAATTTGGGAGGGCCATT
HXT10_A1	TTGACACCTATTTTCGACACTTTT
FLX1_A1	TCTTTTCAGTTTCCTTCCGTTT
PFK26_A4	TTGAATTAGGAAAATTTTGTTGAAGA
RGI2_A1	AAACGGAATCATGGTGGTGT
PET130_A1	ACATCAGCGTAAGCTTTCCA
PET130_A4	TTATACAACCCATAGGAGCAAGAA
YJR030C_A4	TGCGATATATAAGCAATCTCATTT
MDH2_A4	GCCTGCGATGAGCTAAGAAA
(g)	
ACT1_FW	TTGGCCGGTAGAGATTTGAC
ACT1_RS	CCCAAAACAGAAGGATGGAA
MRB1_FW	CAATGAGGAACCCGCTAAAA
MBR1_RS	TGACCTGGGTAGAGAAAACGA
ISF1_FW	CCAGCAAATTCAACGAACAA
ISF1_RS	TTTTCAAGGCCGTATAGATGTG
ALD2_FW	TTGAGGAGGAGCAAGACACA
ALD2_RS	CAACAACGCCAAAAGGAACT
ALD3_FW	TGCAAAGAAGGAGTGGGATG
ALD3_RS	TGGTTGGGGGAATGAAGTAG
ALD4_FW	AGGCCATTACAAACCATCCA

Continuation Table S7	
ALD4_RS	GCACAACAGACCTCACCAGA
ALD5_FW	ACCTACGAACAGCCAACAGG
ALD5_FW	TCTCAACCAAGTCAGCGAGA
ALD6_FW	ACCGTCAACAATTCGACACA
ALD6_FW	GCCATTTCGACACCTTCTTC
